# Impact of Amine Additives on Perovskite Precursor Aging: A Case Study of Light-Emitting Diodes

**DOI:** 10.1021/acs.jpclett.1c01349

**Published:** 2021-06-17

**Authors:** Yan Xu, Weidong Xu, Zhangjun Hu, Julian A. Steele, Yang Wang, Rui Zhang, Guanhaojie Zheng, Xiangchun Li, Heyong Wang, Xin Zhang, Eduardo Solano, Maarten B. J. Roeffaers, Kajsa Uvdal, Jian Qing, Wenjing Zhang, Feng Gao

**Affiliations:** † International Collaborative Laboratory of 2D Materials for Optoelectronics Science and Technology of Ministry of Education, Institute of Microscale Optoelectronics, Shenzhen University, Shenzhen 518060, China; ‡ Department of Physics Chemistry and Biology (IFM), Linköping University, Linköping SE-58183, Sweden; § MACS, Department of Microbial and Molecular Systems, KU Leuven, 3001 Leuven, Belgium; ∥ Key Laboratory for Organic Electronics and Information Displays, Institute of Advanced Materials (IAM), Jiangsu National Synergetic Innovation Center for Advanced Materials (SICAM), Nanjing University of Posts & Telecommunications, 9 Wenyuan Road, Nanjing 210023, China; ⊥ NCD-SWEET beamline, ALBA synchrotron light source, Cerdanyola del Vallès, 08290 Barcelona, Spain; # Guangzhou Key Laboratory of Vacuum Coating Technologies and New Energy Materials, Siyuan Laboratory, Department of Physics, Jinan University, Guangzhou 510632, P. R. China

## Abstract

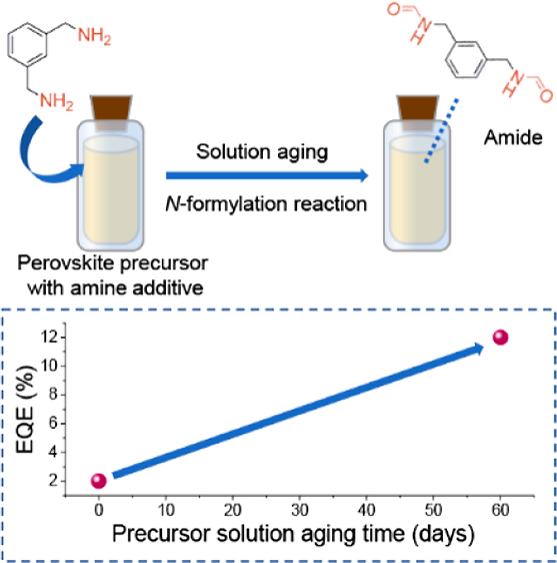

Amines are widely employed as additives for improving the performance of metal halide perovskite optoelectronic devices. However, amines are well-known for their high chemical reactivity, the impact of which has yet to receive enough attention from the perovskite light-emitting diode community. Here, by investigating an unusual positive aging effect of CH_3_NH_3_I/CsI/PbI_2_ precursor solutions as an example, we reveal that amines gradually undergo N-formylation in perovskite precursors over time. This reaction is initialized by hydrolysis of dimethylformamide in the acidic chemical environment. Further investigations suggest that the reaction products collectively impact perovskite crystallization and eventually lead to significantly enhanced external quantum efficiency values, increasing from ∼2% for fresh solutions to ≳12% for aged ones. While this case study provides a positive aging effect, a negative aging effect is possible in other perovksite systems. Our findings pave the way for more reliable and reproducible device fabrication and call for further attention to underlying chemical reactions within the perovskite inks once amine additives are included.

Intense research into metal halide perovskites has led to great advances in solution-processed optoelectronic applications such as photovoltaics, photodetectors, and light-emitting diodes.^1−5^ In addition to the development of various advanced thin-film processing techniques, the remarkable progress in the performance of perovskite optoelectronic devices greatly benefits from compositional engineering.^6−8^ State-of-the-art perovskite inks usually have complex compositions that include not only the necessary combinations of organic and inorganic salts for the construction of perovskite structures but also a wide range of additives for improving device performance.^9−11^ Among others, amines are the most intensively investigated additives in perovskite optoelectronic devices.^12−15^ Various functionalities and advantages, such as defect passivation, crystallization control, and morphological optimization, have been clearly identified.^16−20^ In particular, additive engineering with amines has recently boosted the external quantum efficiency (EQE) values of perovskite light-emitting diodes (PeLEDs) to >20%.^21−23^

In spite of these positive effects, it is well-known that amines are highly reactive and sensitive to heat and light exposure. The high chemical reactivity and poor stability can potentially cause a variety of chemical reactions in the precursor solutions, leading to permanent changes in solution constituents. This is in line with the common observation that the quality of perovskite films is strongly dependent on the shelf storage time of precursor solutions.^24^ Given that additive engineering with amines has become an area of focus in perovskite optoelectronic devices, a detailed understanding of the chemistry within the precursor inks is critically important for reliable and reproducible device fabrication.

Here, we reveal the underappreciated chemical reactivity of amine additives in the precursor solution that significantly affects perovskite crystallization and hence the performance of PeLEDs. We find that N-formylation of amino groups occurs and is accompanied by dimethylformamide (DMF) hydrolysis during solution storage. These reactions are driven and accelerated not only by heating but also by the acidic environment in the solution due to the presence of methylammonium (MA^+^) and/or formamidinium (FA^+^) halides. This gives rise to continuous changes in the constituents of the solution with an increase in shelf storage time and thus varied device performance. Notably, although this behavior may be destructive in most scenarios, we show that the resultant products in CH_3_NH_3_I/CsI/PbI_2_ precursor inks can boost the performance of PeLEDs, resulting in a remarkable EQE enhancement from ∼2% to ∼12%.

We fabricate PeLEDs by subsequent deposition of zinc oxide nanocrystals (ZnO NCs)/polyethylenimine ethoxylated (PEIE)/perovskites/poly(9,9-dioctylfluorene-*co*-*N*-(4-(3-methylpropyl))diphenylamine) (TFB)/MoO_3_/Ag on patterned indium tin oxide (ITO) substrates (Figure 1a). The perovskite emissive layers are deposited by spin-casting the precursor inks with a 1:1.15:1 PbI_2_:CsI:MAI stoichiometry. We select *m*-xylylenediamine (mXDA) (Figure 1a) as the additive with a stoichiometry of 0.6 equiv of lead cations. The PeLEDs prepared from fresh and aged precursor solutions are named control and aged-solution (AS) devices, respectively. Unless otherwise stated, the aged solutions have been stirred at 60 °C for at least 6 days before use.

**Figure 1:**
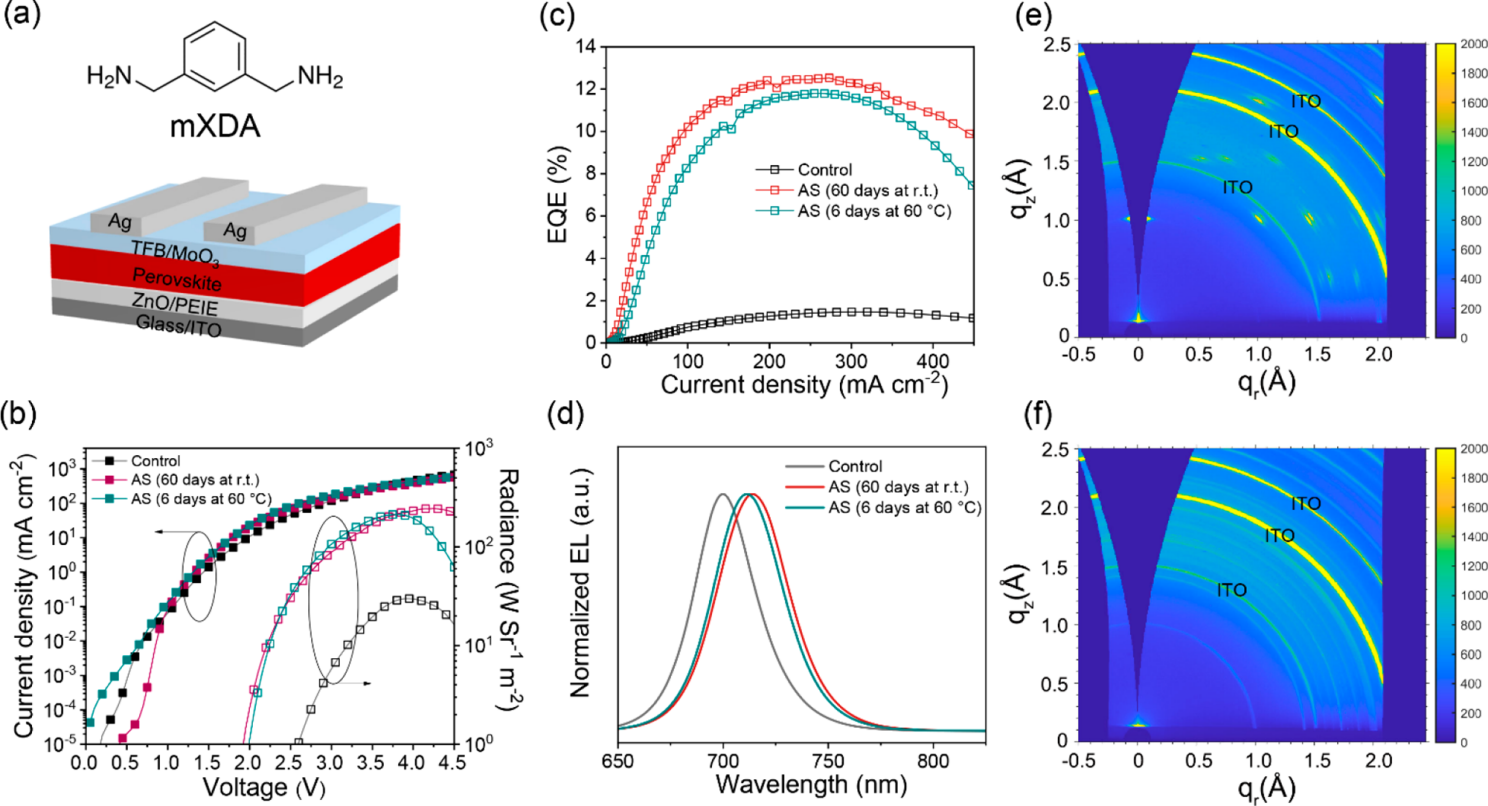
(a) Molecular structure of mXDA and device architecture. Device characteristics for mXDA control devices and aged-solution (AS) counterparts (stirring for 60 days at room temperature and 6 days at 60 °C): (b) current density–voltage–radiance (*J*–*V*–*R*), (c) current density–EQE (*J*–EQE), and (d) EL spectra. Two-dimensional GIWAXS patterns for (e) control and (f) AS perovskite films.

Panels b–d of Figure 1 display the representative characteristics of devices prepared from aged precursors stored at room temperature for 60 days and at 60 °C for 6 days. The control devices prepared from fresh solutions are investigated for comparison. Intriguingly, the aging processes give rise to considerable improvements in the figures of merit of the device in terms of peak EQE values, maximum radiance, and reduced turn-on voltages. Specifically, both AS devices show peak EQEs around 11–12%, which contrasts sharply with those of the control cases showing a low value of ∼2% (Figure 1c). The control devices show EL spectra peaking at 700 nm, a wavelength comparable to the emission from all-inorganic γ-CsPbI_3_. This indicates that MAI is hardly retained in the perovskite films due to their volatility at the high temperature (150 °C for annealing) and the deprotonation ability of the ZnO layer.^25^

Accompanied by the changes in device performance with shelf storage time, the EL peaks shift toward a longer wavelength (from 700 to 716 nm) (Figure 1d). The Tauc plots of the perovskite films (Figure S1) confirm that the EL shifts arise from a bandgap that decreases from 1.74 eV for control films to 1.71 eV for AS cases.

We show the two-dimensional grazing incidence wide-angle X-ray scattering (GIWAXS) patterns of control and AS perovskite films in panels e and f of Figure 1, respectively. Distinct from control films that give discrete Bragg spots, the AS films show randomly distributed crystalline orientations for all respective Debye–Scherrer rings.

Together, the results mentioned above are clearly indicative of continuous compositional variation in the perovskite precursor inks, leading to large differences in the perovskite crystallization process and relevant polycrystalline orientations. These discrepancies ultimately lead to significant variations in the optoelectronic properties of the perovskites and device performance.

To rationalize the chemical processes underlying the positive aging effect of the solutions, the first question that arises is which component(s) in the precursors undergoes chemical reactions during storage. We investigate the organic components closely given that they are chemically less stable and more reactive than the inorganic compounds. First, we independently age MAI and mXDA in DMF and then mix each with the other fresh constituents to make the solutions for device fabrication. The characteristics of representative devices prepared from aged mXDA and fresh MAI or fresh mXDA and aged MAI are summarized in Figure S2. However, no distinct improvement is found here compared to control devices.

We then mix MAI and mXDA in DMF for aging and compare the device performance to that of independently aged MAI and mXDA, aiming to determine whether the chemical processes within the inks involve both components. We show the device characteristics in panels a–c of Figure 2. Aging MAI and mXDA together leads to not only remarkable enhancements in EQE values (reaching ∼12%) but also red-shifted EL spectra (712 nm in this case). In contrast, independently aged MAI and mXDA display moderate performance improvements, where no EL shift is visible compared to that for the use of fresh solutions (Figure 1d).

**Figure 2:**
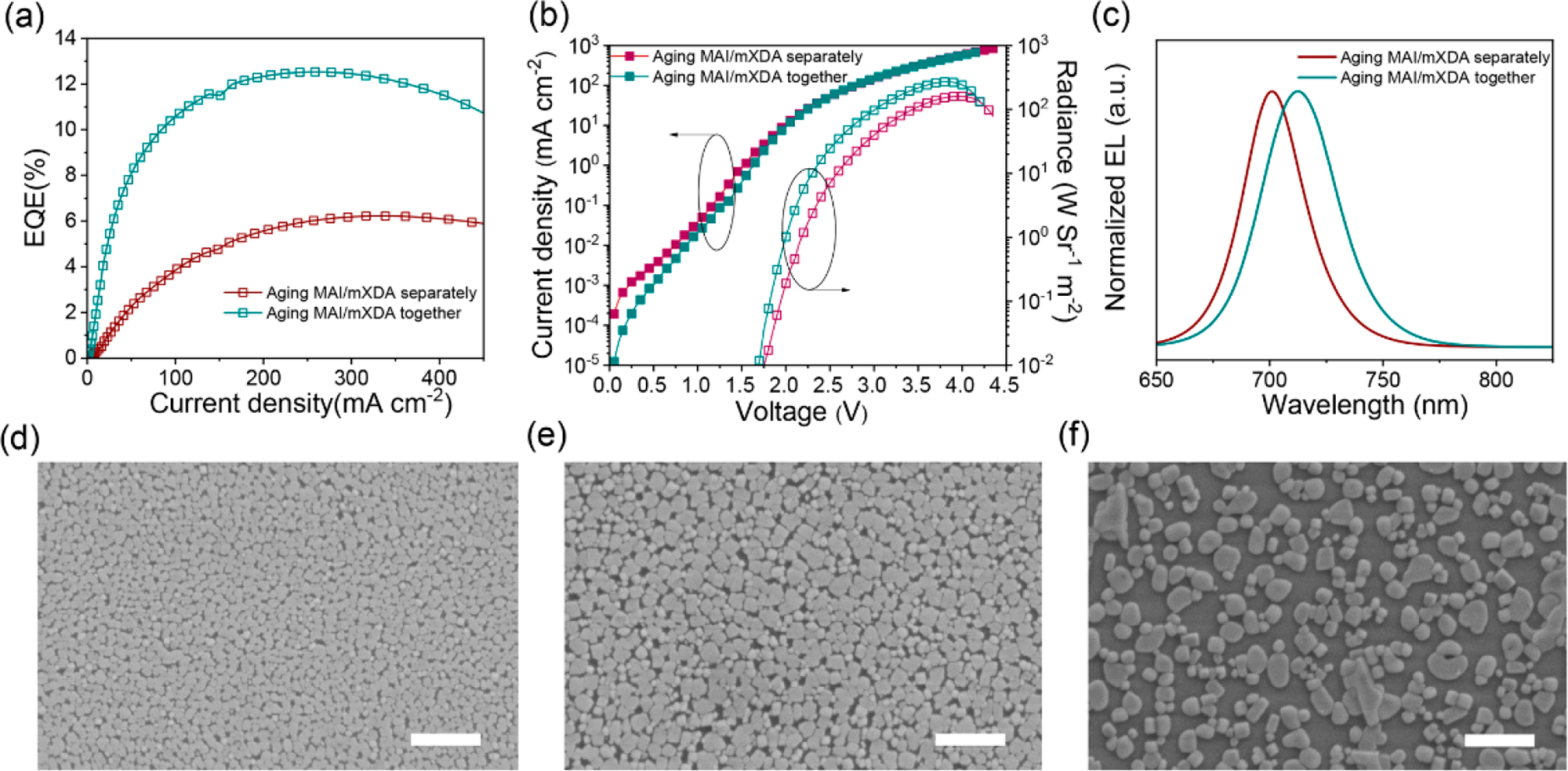
Characteristics for devices prepared from an aged MAI/mXDA mixture and independently aged MA and mXDA in DMF: (a) *J*–EQE, (b) *J*–*V*–*R*, and (c) EL spectra. Scanning electron microscopy images for perovskite films prepared from (d) fresh solutions, (e) separately aged MAI and mXDA solutions, and (f) aged MAI/mXDA mixed solutions. The scale bar is 1 μm.

Consistently, the morphological evolution of the perovskite layers indicates that the underlying chemical processes involve mXDA and MAI simultaneously. The mXDA control films show nanoscale grain sizes and relatively dense surface coverage (Figure 2d). A similar morphology is also visible in the film prepared from independently aged MAI and mXDA, though with slightly larger perovskite grains (Figure 2e). In comparison, aged MAI/mXDA mixtures lead to much larger grain sizes and a discontinuous nanoisland feature (Figure 2f), indicating a significant change in the crystallization process. Previous reports have suggested that the discontinues surface coverage may improve the PeLED performance due to an enhanced light-out coupling efficiency, once the issue of leakage current can be addressed.^22,23^ On the basis of all of the results presented above, we conclude that the chemical process during solution aging is a synergistic effect of MAI and mXDA in DMF, which changes the crystallization process and thus results in distinct improvements in device performance.

We thus investigate the chemical reactions within the MAI/mXDA DMF solutions more closely and perform high-performance liquid chromatography-mass spectrometry (HPLC-MS) to monitor the compositional evolution with storage time. Intriguingly, no signal of mXDA can be detected in the aged samples. Instead, the main components in aged samples show the main fragment with a molecular weight of 193.2, which is 57 units larger than that of mXDA (MW = 136.2) (Figure S3). In addition, to identify whether the reaction(s) involves DMF, we use deuterated DMF as the solvent to prepare the aged MAI/mXDA sample. In this case, the molecular weight of the main product increases to 195.2 (Figure S4), indicating that DMF also participates in the reactions.

In this regard, we infer that the main component detected in aged samples from HPLC-MS is most likely to be protonated *N*-(3-formylaminomethylbenzyl)-formamide (FABF) ([M + H]^+^ = 193.2) as a result of DMF hydrolysis and following N-formylation of mXDA. To verify this, we collect the main products of aged MAI/mXDA samples by preparative liquid chromatography (LC) and then perform nuclear magnetic resonance (NMR) tests. In addition, we synthesize FABF as the reference sample through a well-established N-formylation method.^26^ The synthetic details are summarized in the Supporting Information and Scheme S1. By comparing the ^1^H and ^13^C NMR data as shown in Figure 3a and Figure S5, respectively, we confirm that mXDA undergoes N-formylation reactions with formic acid, which eventually results in the formation of FABF. As mentioned above, MAI is also critical for the reactions within the precursors. Thus, we infer that MA^+^ provides the acidic environment for accelerating DMF hydrolysis, and dimethylamine (DMA) forms as another product.^27^ In addition, the consumption of formic acid and the production of water caused by the N-formylation reaction further promote DMF hydrolysis, facilitating the entire process.

**Figure 3:**
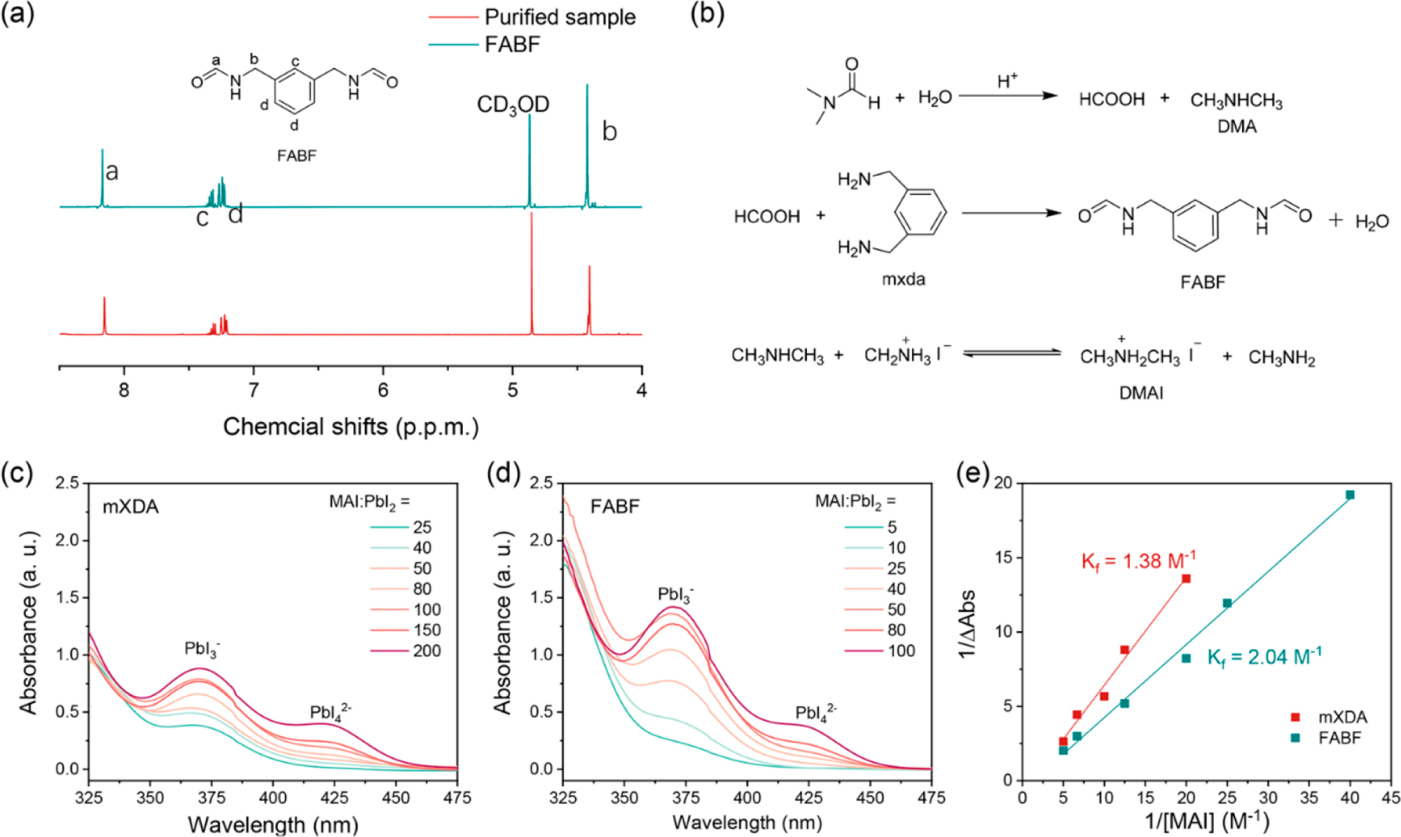
(a) Comparison of ^1^H NMR spectroscopy of purified products from an aged MAI/mXDA DMF solution and FABF (inset). (b) Proposed chemical reactions during precursor aging. Ultraviolet–visible absorbance traces for the different parental solutions with increasing MAI contents: (c) PbI_2_:mXDA and (d) PbI_2_:FABF. (e) BH plots extracted from the traces of PbI_4_^2–^ absorption. The concentrations of PbI_2_ for all of the solutions are 1 mM.

Having revealed the DMF hydrolysis and N-formylation reaction during solution aging, we use the reaction products [FABF and dimethylammonium iodide (DMAI)] as the additives to simulate the eventual components of aged inks and prepare PeLEDs, aiming to further validate our conclusions. We use DMAI because the protonation of *in situ*-formed DMA readily occurs in the presence of excess MAI (Figure 3b). In addition, DMA is in gaseous state at room temperature and hence hard to blend into perovskite inks. The PbI_2_:CsI:MAI:DMAI precursor stoichiometry is 1:1.15:1–*x*:*x*. The mole ratio of FABF is 0.6 equiv to lead cations, which is identical to that of mXDA used in the devices mentioned above.

We note that the formation of FABF is the major reason for morphological evolution during solution aging. As shown in Figure S6a, the FABF/MAI-based films show a nanoisland morphology and large grain sizes like the mXDA AS films. In most scenarios, the changes in film morphology indicate the variations in the crystallization process, which is usually determined by the lead–additive interactions. We thus perform Benesi–Hildebrand (BH) analyses to calculate the formation constant (*K*_f_) of iodoplumbate complex PbI_4_^2–^ in the solutions, by monitoring the evolution of the absorption intensity with an increase in MAI content in PbI_2_/additive parental solutions (Figure 3c,d).^28^ With an increase in MAI content, we observe the gradually increased absorption intensity of PbI_4_^2–^ in both cases. Notably, the PbI_2_/mXDA parental solution requires many more iodide anions to achieve an absorbance comparable to those of FABF cases. We show the BH plots in Figure 3e, from which a smaller *K*_f_ value (1/slope) of 1.37 M^–1^ is observed for mXDA solutions compared to that for the FABF cases (2.04 M^–1^). This indicates that the iodoplumbate complexes are easier to form in FABF solutions compared to the mXDA ones. In other words, mXDA shows a stronger binding affinity with lead cations and hence can hardly be replaced by iodide anions. As such, it serves as a stronger crystalline inhibitor and leads to smaller crystal size and better film coverage, while FABF gives rise to large grain sizes due to its weak ability to inhibit grain growth.^29^ In addition, FABF/DMAI/MAI films (with *x* = 0.4 as an example) give better surface coverage (Figure S6b), suggesting that DMA cations are also involved in perovskite crystallzation.^27,30^

In panels a and b of Figure 4, we show the characteristics of devices with MAI (*x* = 0) and DMAI/MAI (*x* = 0.4) as the organic components. We note that using FABF alone is enough to remarkably improve the peak EQE to 9.6%. Upon replacement of some MAI with DMAI (*x* = 0.4), the peak EQE value can be further enhanced to 12.1%, which is as high as that of the mXDA AS devices shown in Figure 1. The optimized devices show negligible current efficiency roll-off, with no obvious EQE decrease until a large current density of ∼700 mA cm^–2^. This gives rise to a large radiance of 434 W sr^–1^ m^–2^.

**Figure 4:**
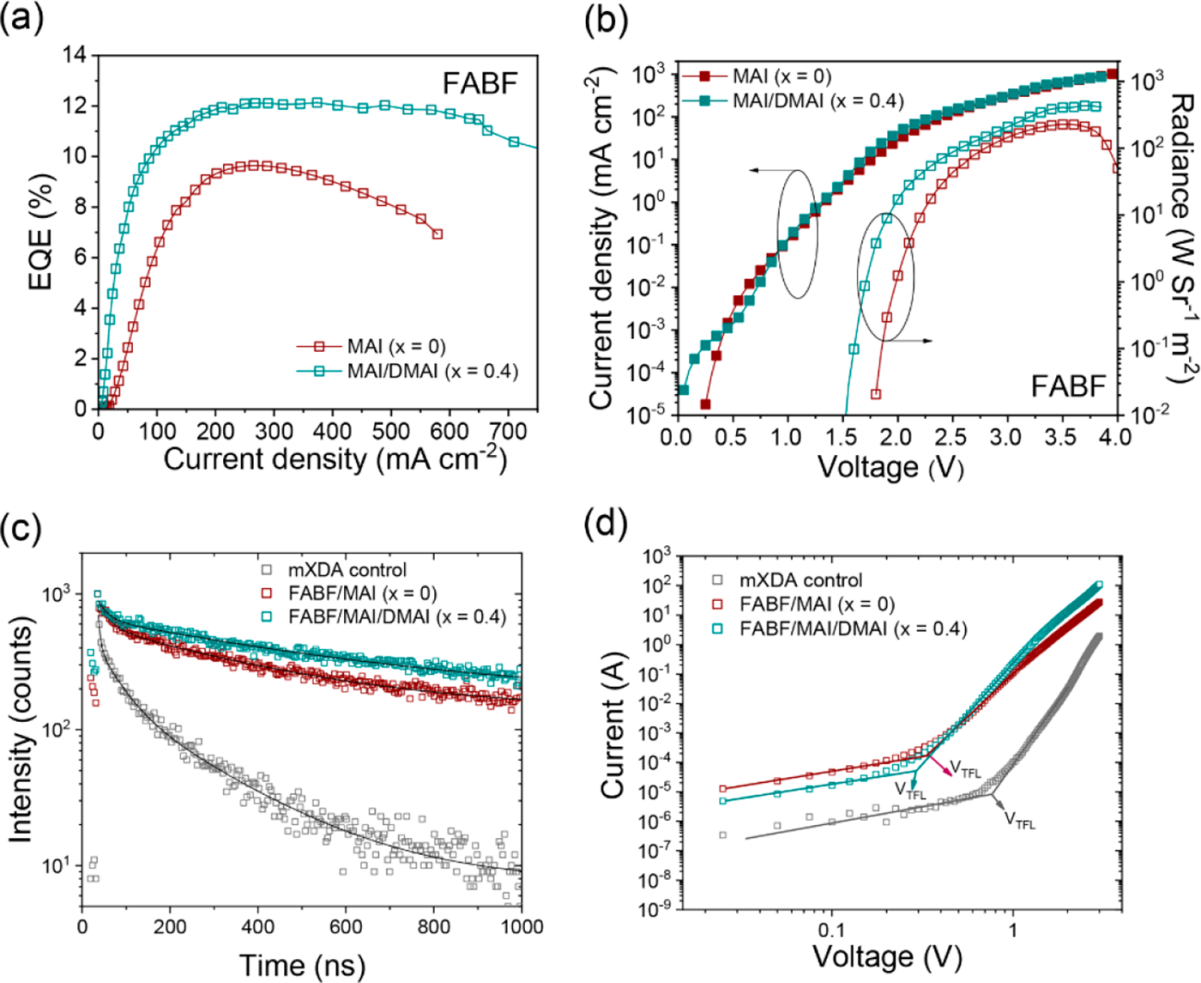
Characteristics for FABF devices with MAI and MAI/DMAI (*x* = 0.4) as the organic cations: (a) *J*–EQE and (b) *J*–*V*–*R*. (c) PL decay traces of mXDA control films and that prepared with FABF/MAI and FABF/MAI/DMAI (*x* = 0.4) mixtures. (d) Determination of *V*_TFL_ for probing trap density in the devices.

The performance enhancement can be attributed to reduced nonradiative recombination pathways, which is first evident by the remarkably prolonged PL lifetime as confirmed by time-correlated single-photon counting (TCSPC) measurements (Figure 4c). To further evaluate the discrepancies in the densities of traps (*N*_dt_) within the devices, we measure the *J*–*V* curves of electron-only devices in the dark, with an ITO/ZnO/PEIE/perovskites/[6,6]-phenyl-C_61_-butyric acid methyl ester (PC_60_BM)/LiF/Al architecture. The representative characteristics are shown in Figure 4d, from which we observe a linear relation at low bias voltage followed by a nonlinear rise. The former corresponds to the ohmic response, and the latter is assigned to the trap-filled limit regime where *J* ∝ *V^n^* (*n* > 3). From the kink point (*V*_TFL_), we deduce the value of *N*_dt_ according to the following equation:
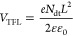
where *L* is the film thickness, *e* is the elementary charge, ε is the dielectric constant, and *ε*_0_ represents the vacuum permittivity. The average trap densities determined from three devices are 1.5 × 10^16^, 6.4 × 10^15^, and 5.9 × 10^15^ cm^–3^ for mXDA control films, FABF/MAI films, and FABF/MAI/DMAI films, respectively. In addition to further reducing the extent of charge trapping, we note that the addition of DMAI can effectively improve the charge injection, as suggested by the *J*–*V* curves of the single-carrier devices. It could be one of the reasons for the high radiance and mitigated current efficiency roll-off in the optimized FABF/MAI/DMAI devices (*x* = 0.4). As such, we conclude that the performance enhancements in mXDA devices with prolonged solution aging times are collectively caused by the formation of FABF and DMAI, leading to mitigated trap-assisted nonradiative recombination and more efficient charge injection.

Notably, the EL spectra shift toward the longer wavelength with an increase in DMAI content [from *x* = 0 to 1 (Figure 5a)], analogous to the bandgap evolution observed in mXDA devices with prolonged solution aging time. To identify whether FABF also plays a key role in determining the bandgap, we prepare perovskite films without using FABF, that is, with a 1:1.15:1:0.6 DMAI:CsI:PbI_2_:mXDA stoichiometry. As shown in Figure S7, the optical bandgap determined by the Tauc plot is 1.74 eV and the PL peak remains at 700 nm. In this scenario, the bandgap evolution of the perovskites during solution aging is collectively caused by the production of both DMAI and FABF.

**Figure 5:**
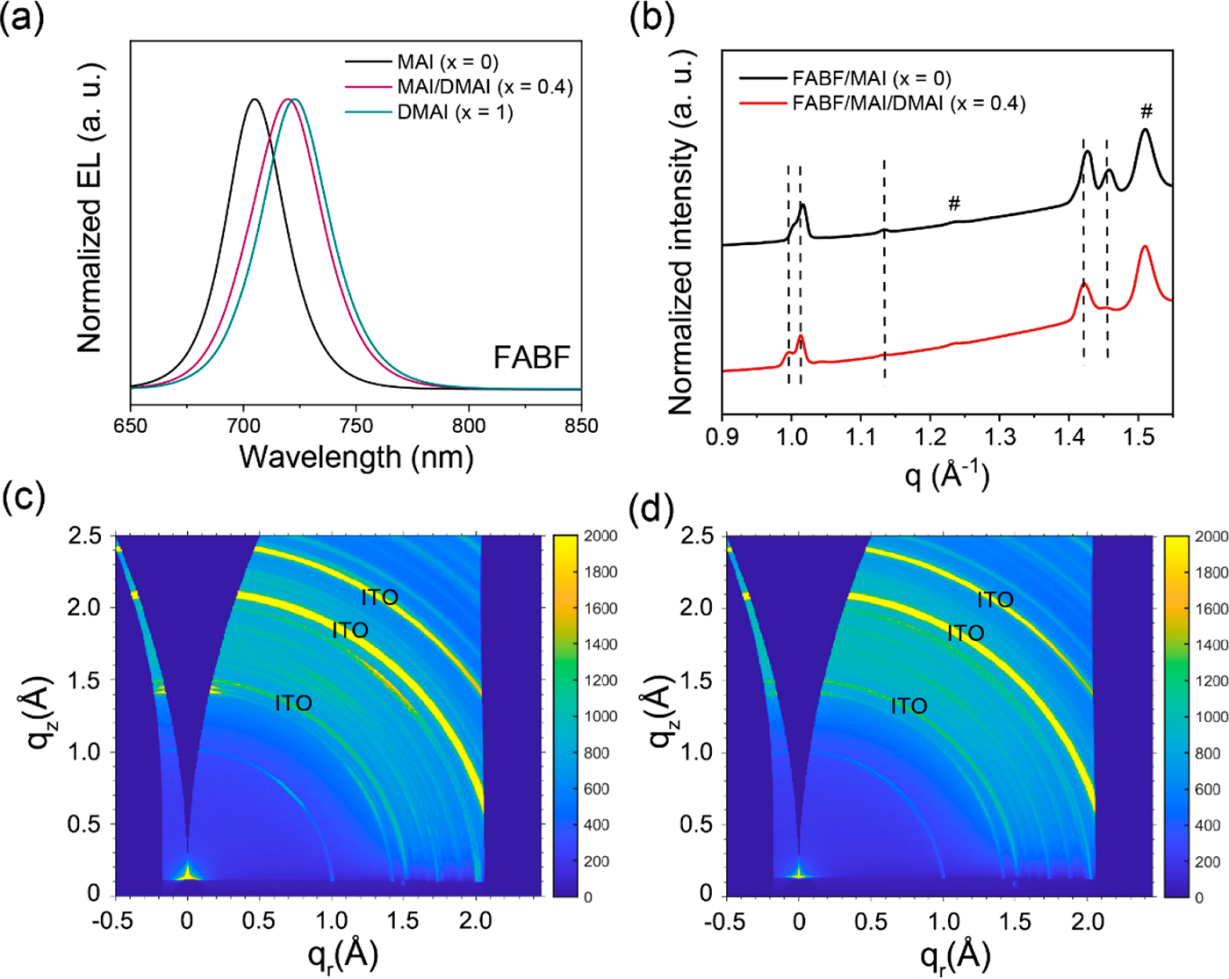
(a) EL spectra for FABF devices with MAI (*x* = 0), MAI and DMAI (*x* = 0.4), and DMAI (*x* = 1) in the precursor solution. 2D GIWAXS measurements for probing the crystal structure: (b) integrated scattering intensity profiles and 2D GIWAXS patterns for perovskite films prepared from (c) FABF/MAI and (d) FABF/MAI/DMAI (*x* = 0.4) precursor solutions. Here, the # symbols in panel b denote the diffraction peaks from ITO.

One possible explanation is that FABF and DMA cations together induce a shift in the crystal lattice symmetry and thus lead to a variation in the bandgap. Similar observations can be found in recent work on perovskite photovoltaics where DMAI is used to stabilize β-CsPbI_3_ at room temperature with a reduced bandgap.^30−32^ We thus complete GIWAXS to characterize the crystal structure of FABF/MAI films with optimized DMAI addition (*x* = 0.4). We show the integrated X-ray scattering intensity profiles with *q* values ranging from 0.9 to 1.55 Å in Figure 5b and two-dimensional (2D) GIWAXS patterns in panels c and d of Figure 5. The whole scattering patterns and their corresponding structural refinements are shown in Figure S8a, from which we note that all of the diffraction peaks of FABF/MAI films can be well assigned to orthorhombic γ-CsPbI_3_. In comparison, small shifts in angles are visible in FABF/MAI/DMAI samples (Figure 5b and Figure S8a), indicating an expansion of the crystal lattice (Figure S8b). We also notice some differences in peak splitting. These variations confirm changes in the octahedral tilts and lattice distortions in the γ-CsPbI_3_ structure. Further analyses of the spontaneous strain suggest that the use of FABF/DMAI films mitigates the distortions in the perovskite crystals, reducing the tilting of γ-phases and hence making them more tetragonal-like (Figure S8c).^33,34^ These changes are also in line with the discrepancy of GIWAXS patterns between mXDA control and AS films (Figure S9).^33^ As such, we conclude that the gradual formation of DMAI and FABF in the aged precursors collectively make the orthorhombic γ-CsPbI_3_ phases more tetragonal-like and hence lead to the bandgap variations.

To generalize our findings, we investigate the aging behavior in other material systems. We first employ 4,7,10-trioxa-1,13-tridecanediamine (TTDDA) as the additive and prepare the devices with the respective fresh and aged solutions (with a 1:1.15:1 PbI_2_:CsI:MAI stoichiometry).^21,23^ Consistent with the aromatic amines (mXDA) mentioned above, the precursors with aliphatic amines show identical positive aging behavior, with the aged solution showing red-shifted EL emission and much better performance (Figure S10). In addition, we demonstrate that N-formylation of amines also occurs with FA^+^-involved perovskite precursors, which is confirmed by the formation of FABF in an FAI/mXDA mixed DMF solution as indicated by HPLC-MS results (Figure S11). All of these results suggest that N-formylation widely occurs in amine-involved perovskite precursors. It is worth mentioning that in our previous work about TTDDA-passivated near-infrared and blue perovskite emitters, only a fresh precursor solution gives decent performance.^21,23^ We thus believe that N-formalization of amine additives does not always improve device performance; instead, it is a critical issue leading to varied device performance. Our findings thus call for further studies of device fabrication once amine additives are used.

In summary, we have revealed that the widely employed amine additives readily undergo N-formylation, accompanied by hydrolysis of DMF in perovskite precursor solutions, leading to continuous changes in the constituents with storage time and thus varied device performance. In particular, these reactions give rise to the positive aging phenomenon in CH_3_NH_3_I/CsI/PbI_2_ precursor solutions in which the performance of light-emitting diodes improves with solution storage time. Our results show that the N-formylation and hydrolysis products collectively impact perovskite crystallization, resulting in a reduced trap density and the transition from orthorhombic γ-CsPbI_3_ to more tetragonal-like phases. These effects hence lead to improved electroluminescence performance and red-shifted emissions. Our results not only provide a useful strategy for fabricating deep-red CsPbI_3_ light-emitting diodes with decent performance but also uncover the hidden effects of chemical reactivity of amine additives on perovskite precursor solutions.

## Supporting Information Available

Experimental details, supplementary scheme for material synthesis, supplementary figures for material and device characteristics, and supplementary references (PDF)
