# Supplementary material for: Impact of Amine Additives on Perovskite Precursor Aging: A Case Study of Light-Emitting Diodes
Source: J Phys Chem Lett. 2021 Jun 17;12(25):5836–43. doi: 10.1021/acs.jpclett.1c01349 (PMC8256416; doi:10.1021/acs.jpclett.1c01349)
Supplement: Supplementary file 1 — jz1c01349_si_001.pdf [file jz1c01349_si_001.pdf]

Supporting Information

The Impact of Amine Additives on Perovskite Precursor Aging: A Case Study on Light Emitting Diodes

Yan Xu,[a,b] Weidong Xu,[b] Zhangjun Hu,[b] Julian A. Steele,[c] Yang Wang,[d] Rui Zhang,[b] Guanhaojie Zheng,[b] Xiangchun Li,[d] Heyong Wang,[b] Xin Zhang,[b] Eduardo Solano,[e] Maarten B. J. Roeffaers,[c] Kajsa Uvdal,[b] Jian Qing,[b,f] Wenjing Zhang,[a]* and Feng Gao[b]**

[a] International Collaborative Laboratory of 2D Materials for Optoelectronics Science and Technology of Ministry of Education Institute of Microscale Optoelectronics, Shenzhen University, Shenzhen 518060, China.

[b] Department of Physics Chemistry and Biology (IFM), Linköping University, Linköping SE-58183, Sweden.

[c] cMACS, Department of Microbial and Molecular Systems, KU Leuven, 3001 Leuven, Belgium.

[d] Key Laboratory for Organic Electronics and Information Displays, Institute of Advanced Materials (IAM), Jiangsu National Synergetic Innovation Center for Advanced Materials (SICAM), Nanjing University of Posts & Telecommunications, 9 Wenyuan Road, Nanjing 210023, China.

[e] NCD-SWEET beamline, ALBA synchrotron light source, 08290, Cerdanyola del Vallès, Barcelona, Spain

[f] Guangzhou Key Laboratory of Vacuum Coating Technologies and New Energy Materials, Siyuan Laboratory, Department of Physics, Jinan University, Guangzhou 510632, P. R. China.

* Email: weidong.xu@liu.se; wjzhang@szu.edu.cn; feng.gao@liu.se

Experimental Procedures

Materials: m-xylylenediamine (mXDA, 99%), 4,7,10-trioxa-1,13-tridecanediamine (TTDDA, 99%), dimethylammonium iodide (DMAI, 99.9%), caesium iodide (CsI,99.999%) and ammonium formate were purchased from Sigma-Aldrich. Methylammonium iodide (MAI) and formamidinium iodide (FAI) were purchased from GreatCell Solar. Ultradry lead iodide (PbI_2, 99.999%) was purchased from Alfa Aesar. Poly(9,9-dioctyl-fuorene-co-N-(4-butylphenyl)diphenylamine) (TFB) was purchased from American Dye Source. Other materials for device fabrication and materials synthesis were all purchased from Sigma-Aldrich. ZnO nanoparticles were synthesized as reported in the literature.[1]

Synthesis of N,N'-(1,3-phenylenebis(methylene))diformamide (FABF): m-xylylenediamine (273 mg, 2.0 mmol), ammonium formate (126 mg, 4.0 mmol) were dissolved in ultradry CH_3CN (30 mL) in a degassed vessel. The mixture was heated at 100 °C for 24 h. After cooling down, CH_3CN was removed. The residue was washed by water and then extracted by ethyl acetate (30 mL) for three times. The organic phase was combined and dried over $MgSO_4$. After the solvent was removed, the residue was recrystallized using methyl/dichloromethane (1:8) and washed by dichloromethane three times to give FABF as a white solid (326 mg, 84.9%). The ^1H and ^{13}C NMR spectra were recorded on a Varian 500 MHz NMR spectrometer. ^1H NMR (500 MHz, CD_3OD): δ 8.16 (s, 2H), 7.35-7.30 (m, 1H), 7.26-7.21 (m, 3H), 4.43-4.40 (d, 4H). ^{13}C NMR (126 MHz, CD_3OD): δ 162.23, 138.49, 128.48, 126.38, 126.18, 41.16.

High-performance liquid chromatography-mass spectrometry (HPLC-MS) and preparative liquid chromatography: The HPLC-MS analysis was run on a Waters system equipped with Waters 525 gradient pumps, 2998 Photodiode Array Detector, 2424 Evaporative Light Scattering Detector, SQD 2 Mass Detector, and an XBridge C18 column (4.6 × 50 mm, 3.5 mm). With the flow rate of 1.5 mL/min, a

binary linear gradient of A/B 100:0 to 70:30 over 4 min followed by an additional 2 min at 0:100 was used, where A: 95:5 water/acetonitrile buffered with 10 mM NH_4OAc at pH 6.8–7.0, and B: 90:10 acetonitrile/water buffered with 10 mM NH_4OAc at pH 6.8−7.0. Preparative liquid chromatography was run on the same Waters system but in preparative mode with an XSelect phenyl-hexyl column (19 × 250 mm, 5.0 μm). With the flow rate is 25 mL/min, a binary linear gradient of A and B was used. The simple retreatments of aged samples were conducted prior to HPLC-MS analysis. Each aged sample was poured into the water, and subsequently extracted by ethyl acetate and washed by brine to get rid of the excess DMF and MAI or FAI. A proper portion of the organic phase was used for the determination and preparation.

Thin-film Characterizations: Ultraviolet-visible (UV-vis) absorbance spectra were collected from a PerkinElmer Lambda 900. Morphological images of the perovskite films were measured by a scanning electron microscope (SEM, LEO 1550 Gemini) under a voltage of 3 kV. Time-correlated single photon counting (TCSPC) for PL lifetime was measured by using an Edinburgh instruments spectrometer (FLS1000) with a 405 nm picosecond laser (less than 100 ps, 1 MHz). The instrument response function (IRF) is less than 200 ps.

Grazing incident wide-angle X-ray scattering (GIWAXS). GIWAXS data were obtained from NCD-SWEET beamline, ALBA Synchrotron, Spain. The energy of the X-ray beam was set to 12.95 keV using a Si (1 1 1) channel-cut monochromator and further collimated with an array of Be lenses. The incidence angle was 1° and the diffraction patterns were collected using a Rayonix® LX255-HS area detector, which consist of a pixel array of 5760 × 1920 (V × H) with a pixel size of 88.54 × 88.54 μm^2 for the pixel binning employed of 2 × 2. The scattering vector q was calibrated using Cr_2O_3 as standard, obtained using a sample to detector distance of 220 mm. All the samples were measured under N_2 atmosphere.

Preparation of perovskite solution. Perovskite precursor inks were prepared with a stoichiometry of PbI_2: CsI: MAI: mXDA = 1: 1.15: 1 :0.6 in dimethylformamide (DMF). Unless otherwise stated the perovskite inks were stirred at 60°C for at least 6 days before use to finish the *N*-formylation reaction. For FABF added precursors, a stoichiometry of PbI_2: CsI: MAI: DMAI is 1: 1.15: $1-x$: x was used. The mole ratio of FABF is 0.6 equivalent to Pb^{2+} cations. The optimal x value is 0.4. The optimized concentration for lead cations is 0.12 M.

UV-vis absorbance spectra for the solutions and Benesi−Hildebrand (BH) analyses. Ultraviolet-visible (UV-vis) absorbance spectra for solutions were collected by a PerkinElmer Lambda 35 at room temperature. Anhydrous DMF was used as the solvent. The concentration of Pb^{2+} for all the solutions is fixed at 1 mM. The mole ratio of PbI_2: mXDA and PbI_2: FABF parent solution is 1:5 for both cases. The formation equilibrium constant (K_f) of PbI_4^{2-} was calculated by the Benesi−Hildebrand equation stated below:

Device fabrication. Patterned ITO glass substrates were sequentially cleaned by TL-1 (a mixture of water, ammonia (25%) and hydrogen peroxide (28%) (5:1:1 by volume) and deionized water, followed by UV-ozone treatment for 15 min before use. After cooling to the room temperature, ZnO nanoparticles were spin-coated onto ITO substrates at 4000 r.p.m. for 30 s in ambient. Next, the ZnO-coated substrates were moved into a N_2-filled glove box to deposit PEIE (1.1 mg mL^{-1} in isopropanol) at 5000 r.p.m. for 30 s, followed by annealing at 100 °C for 10 min. After cooling down, perovskite precursors were spin-coated at 4000 r.p.m. for 30 s and then annealed on a pre-heated hotplate at 150 °C for 5 min. TFB layer (12 mg mL^{-1} in chlorobenzene) was deposited on top of the perovskite films at 2000 r.p.m. for 30 s. Finally, 7 nm MoO_3 and 100 nm Ag were deposited as the contacts in a thermal evaporator, with the deposition rates of ~0.2 and ~1.5 Å s^{-1} respectively. The active area of our PeLEDs is 7.25 mm^2.

Single carrier devices and determination of trap density (N_{dt}). The electron only devices were fabricated with an architecture of ITO/ZnO/PEIE/perovskite/$PC_{60}BM$/LiF/Al. All the concentration for lead cations of perovskite precursor solution is 0.5 M, giving a thick layer of ~300 nm for all the case. The $PC_{60}BM$ layer was deposited on top of perovskite at 3000 r.p.m. for 30 s, followed by thermal annealing at 70 °C for 15 min. 1 nm LiF and 120 nm Al were deposited by a thermal evaporator, with the deposition rates of ~0.1 and ~5 Å s^{-1} respectively. The current-voltage (J-V) curves were scanned from 0 to 3 V with a step of 0.025 V. The trap density was calculated by the following equation:

$$V_{TFL} = \frac{eN_{dt}L^2}{2\varepsilon\varepsilon_0}$$

Where L is the film thickness, e and ε_0 represent the elementary charge and vacuum permittivity, respectively. ε is the dielectric constant of $CsPbI_3$. The ε is 19.2 as recorded from the literature.[2]

Device Characterization. The PeLEDs were measured at room temperature in a N_2-filled glove box. A Keithley 2400 source meter incorporated with a QE Pro spectrometer (Ocean Optics) was used to measure the electrical output of PeLEDs. The applied voltage started from 0 V and increased with a step of 0.05 V, lasting for 300 ms at each voltage step for stabilization and measurements. The PeLEDs are tested on top of the integration sphere and only forward light emission can be collected, consistent with the standard OLED characterization method. The setup is calibrated by a standard Vis–NIR light source from Ocean Optics (HL-3P-INT-CAL plus).

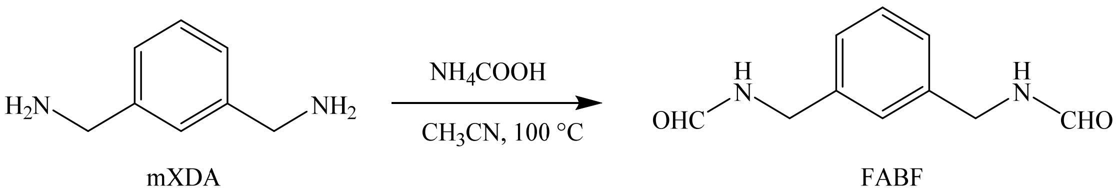

Scheme S1. The synthetic route of FABF.

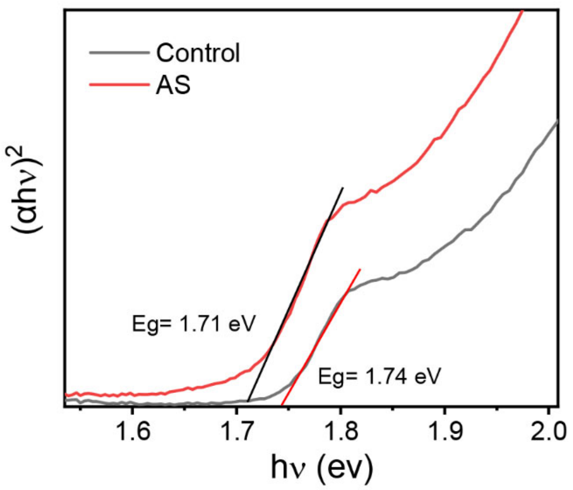

Figure S1. Tauc plots of mXDA control and AS films.

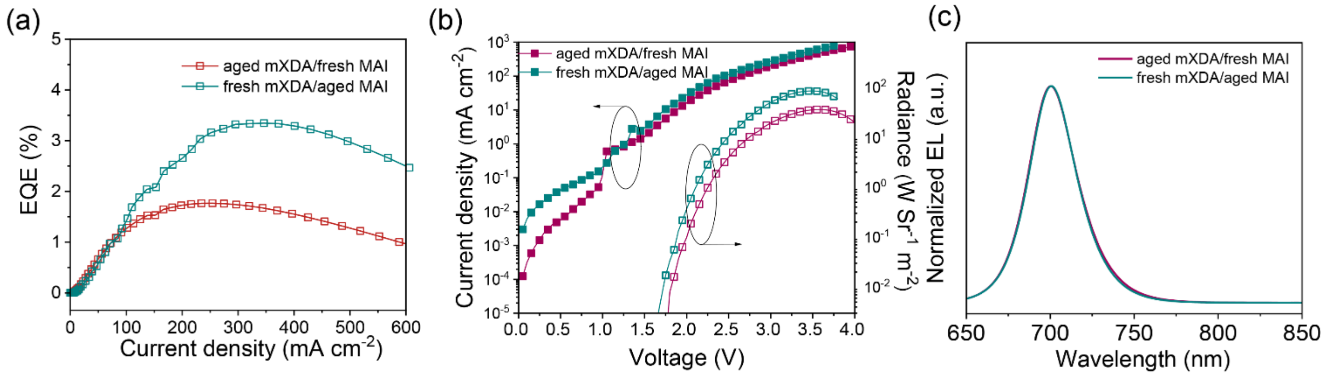

Figure S2 Representative characteristics of PeLED devices prepared from the precursor solution with aged mXDA/fresh MAI and fresh mXDA/aged MAI respectively. a) Current density-EQE (J-EQE). b) Current density-voltage-radiance (J-V-R). c) EL spectra.

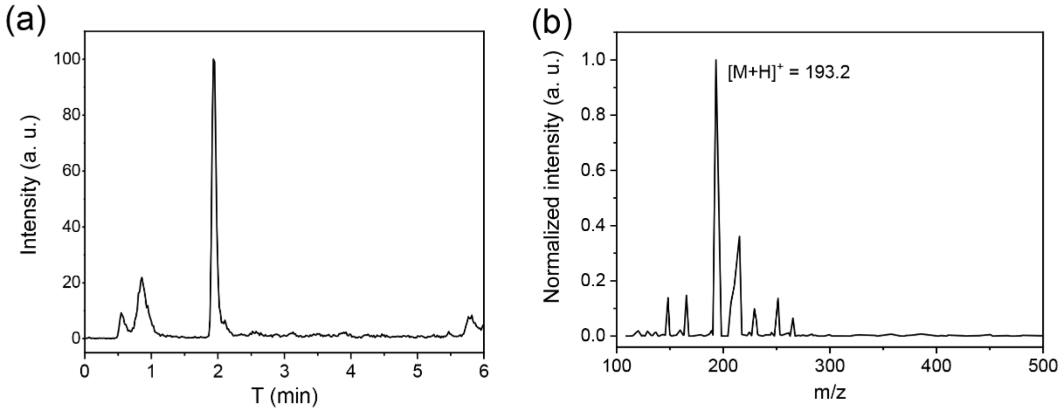

Figure S3 a) HPLC chromatography for aged mXDA/MAI sample. b) Mass spectrometry for the main product collected by HPLC (with retention time of around 2 min).

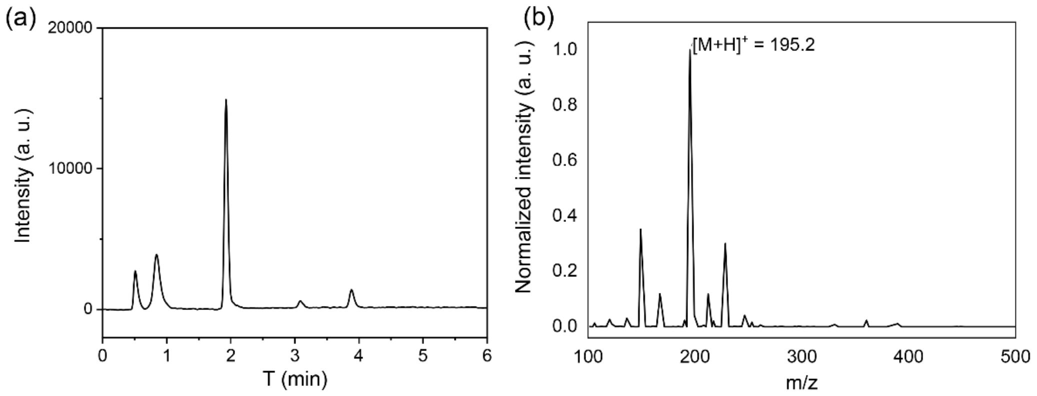

Figure S4 a) HPLC chromatography for aged mXDA/MAI sample. b) Mass spectrometry for the main product collected by HPLC (with retention time of around 2 min). Here, deuterated DMF was used as the solvent for dissolving mXDA and MAI.

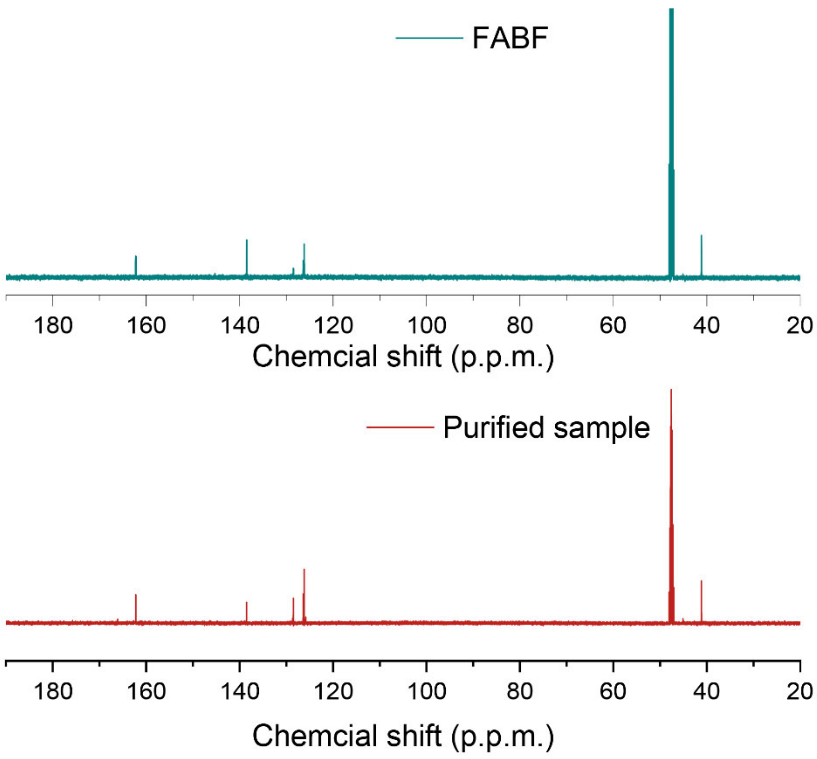

Figure S5 ^{13}C NMR spectra of synthesized FABF and purified sample from aged perovskite precursor solution. Here, deuterated methanol (CD_3OD) is used as the solvent.

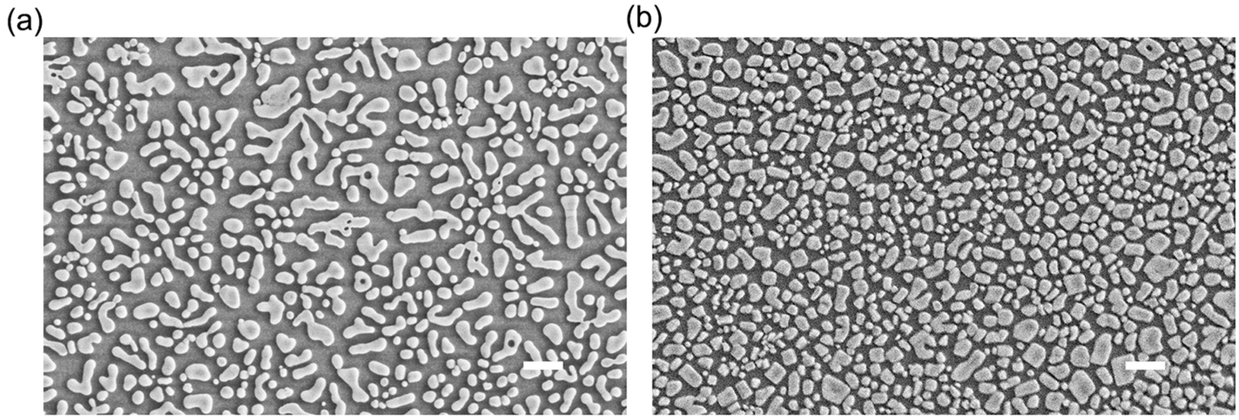

Figure S6 SEM topography images for a) FABF/MAI-based perovskite thin films; b) FABF/MAI/DMAI (x = 0.4) perovskite films. All the films were prepared on ITO/ZnO:PEIE substrates. The scale bars are 1 μm.

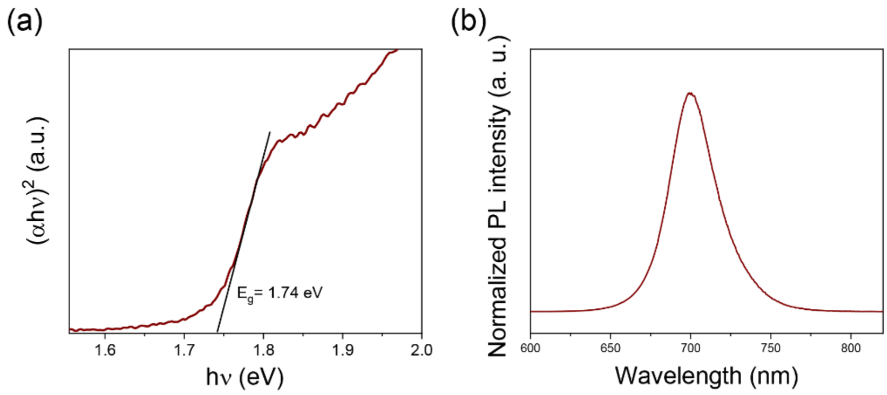

Figure S7 a) Tauc plot for determining the bandgap of mXDA/DMAI perovskite films. b) PL spectrum of mXDA/DMAI perovskite films. Here the stoichiometry for the perovskite precursor solution is DMAI: CsI: PbI2: mXDA = 1: 1.15: 1: 0.6.

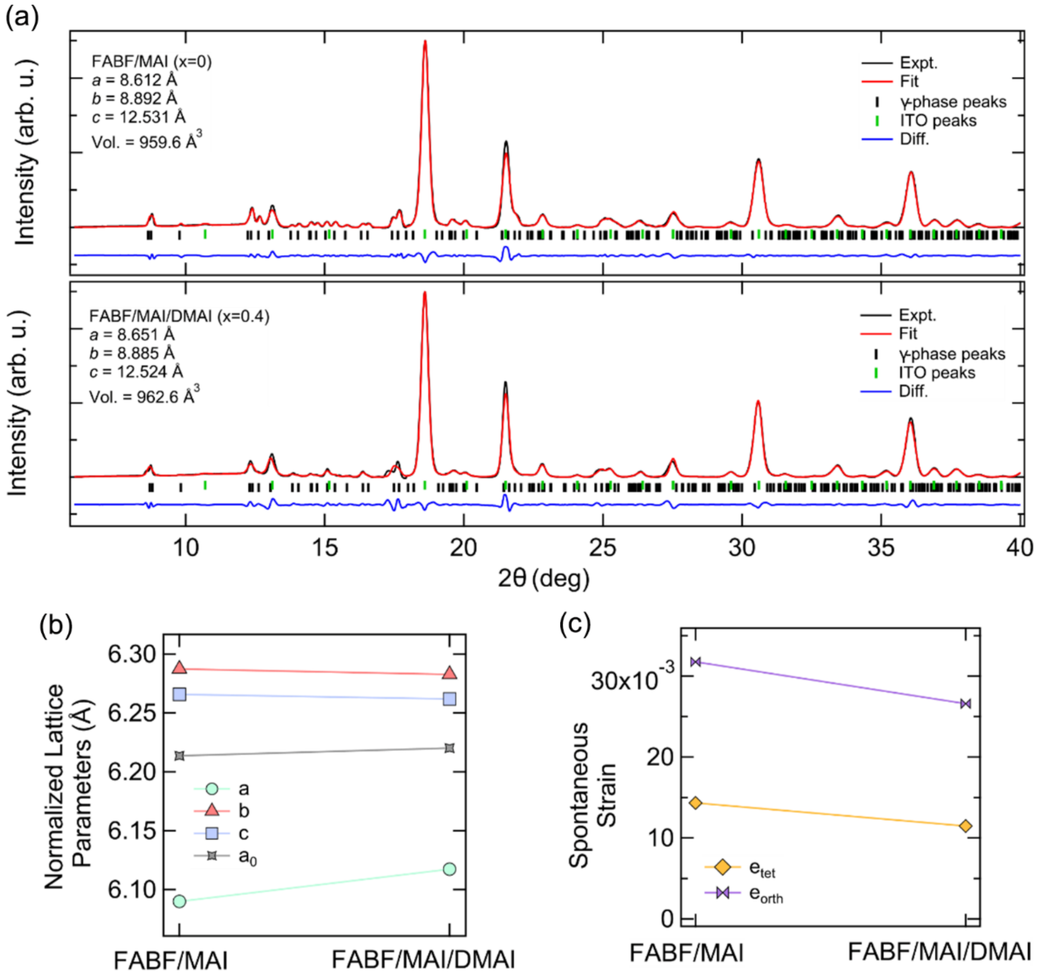

Figure S8: a) Synchrotron GIWAXS patterns and their structural refinements (La Bail method; λ = 0.9574 Å) of γ-$CsPbI_3$ thin films. Derived lattice parameters are presented on the left. For clarity, the scattering data have been background corrected and offset. b) Normalized lattice parameters of γ-$CsPbI_3$ perovskite with and without DMAI. Normalization of the γ-phase lattice is made via rescaling the orthorhombic unit cell by (1/√2, 1/√2, 1/2). c) Decoupled orthorhombic (e_{orth}) and tetragonal (e_{tet}) spontaneous strain components.

For phase transitions in which the high symmetry α-phase is reduced to a degenerate γ-phase, the number of distortion components can be expressed in terms of symmetry-adapted strains (ref. 11c in the main text). The symmetry-breaking distortions are thus divided into the tetragonal (e_{tet}) and orthorhombic (e_{orth}) strains, manifesting the β-phase and γ-phase, respectively.[3] These quantities are calculated relative

to an undistorted cubic unit cell, a_0, which is estimated by taking the cube root of the normalized unit cell volume. It follows that the spontaneous strain components are defined as: $e_1 = (a - a_0)/a_0$, $e_2 = (b - a_0)/a_0$ and $e_3 = (c - a_0)/a_0$, where a, b and c are the normalized lattice parameters of the $CsPbI_3$ orthorhombic phase. The separate strain components contributing to the lattice distortions are then given here by: $e_{orth} = e_2 - e_1$ and $e_{tet} = (2e_3 - e_1 - e_2)/\sqrt{3}$. A factor of $\sqrt{3}$ is included here to ensure that the two strains are on the same scale.

Starting from FABF/MAI perovskite, this material aligns its crystal symmetry with other reports. The inclusion of DMAI suppresses the spontaneous strains e_{orth} and e_{tet} by roughly 20% and 16%, respectively, reducing octahedral tilting and shifts the crystal symmetry toward a tetragonal-like system (i.e. β-phase).

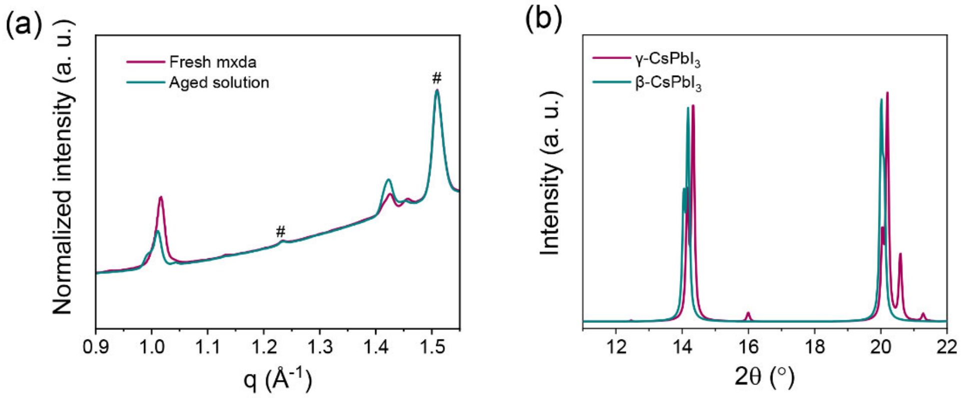

Figure S9 a) The patterns of integrated diffraction intensity verse q values extracted from GIWAXS measurements shown in Fig. 1 (mXDA control and AS films). Here, # diffraction peaks from ITO. b) The X-ray diffraction (XRD) patterns (λ = 0.413906 Å) for β-$CsPbI_3$ and γ-$CsPbI_3$ from Marronnier et al.[4]

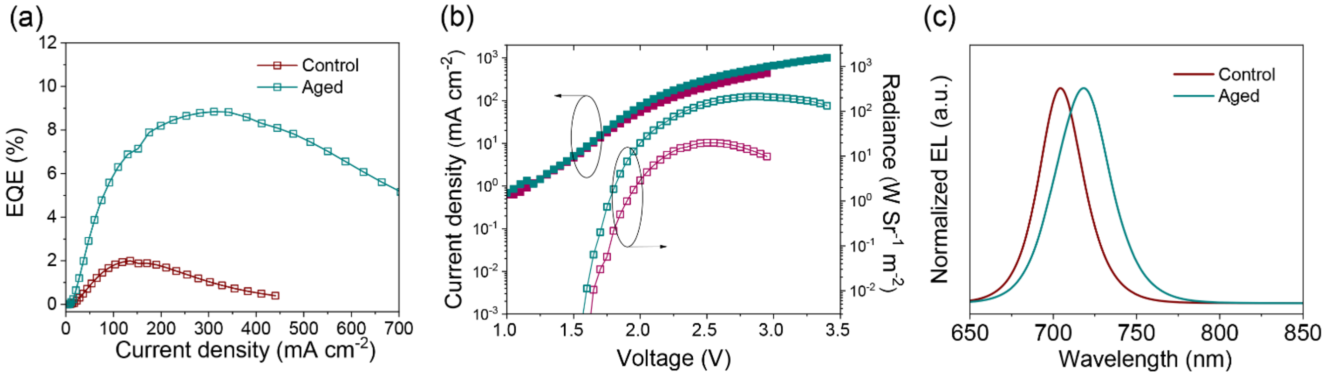

Figure S10 The representative characteristics of TTDDA based PeLED devices prepared with fresh (control) and aged solutions. a) Current J-EQE. b) J-V-R. c) EL spectra.

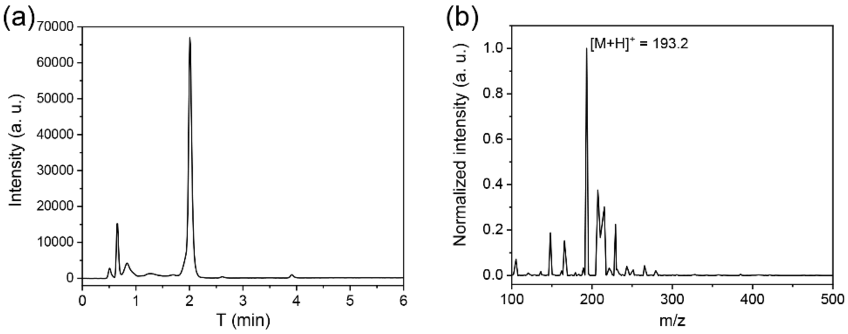

Figure S11 HPLC chromatography (a) and MS spectrum (b) of mXDA/FAI solution after aging.

References

(1) Schwartz, D. A.; Norberg, N. S.; Nguyen, Q. P.; Parker, J. M.; Gamelin, D. R., Magnetic Quantum Dots: Synthesis, Spectroscopy, and Magnetism of Co^{2+}- and Ni^{2+}-Doped ZnO Nanocrystals. *J. Am. Chem. Soc.* **2003,** *125*, 13205-13218.

(2) Schlaus, A. P.; Spencer, M. S.; Miyata, K.; Liu, F.; Wang, X.; Datta, I.; Lipson, M.; Pan, A.; Zhu, X. Y., How lasing happens in $CsPbBr_3$ perovskite nanowires. *Nat. Commun.* **2019,** *10*, 265.

(3) Steele, J. A.; Lai, M.; Zhang, Y.; Lin, Z.; Hofkens, J.; Roeffaers, M. B. J.; Yang, P., Phase Transitions and Anion Exchange in All-Inorganic Halide Perovskites. *Acc. Mater. Res.* **2020,** *1*, 3-15.

(4) Marronnier, A.; Roma, G.; Boyer-Richard, S.; Pedesseau, L.; Jancu, J. M.; Bonnassieux, Y.; Katan, C.; Stoumpos, C. C.; Kanatzidis, M. G.; Even, J. Anharmonicity and Disorder in the Black Phases of Cesium Lead Iodide Used for Stable Inorganic Perovskite Solar Cells. *ACS Nano* **2018,** *12*, 3477-3486.
